# Short Synthetic Lipopeptides C_16_-KK-NH_2_ and (C_10_)_2_-KKKK-NH_2_ Enhance the Activities of Erythromycin and Tetracycline Against ESKAPE Pathogens

**DOI:** 10.3390/antibiotics15050439

**Published:** 2026-04-28

**Authors:** Malgorzata Anna Paduszynska, Alena Shchuka, Katarzyna Ewa Greber

**Affiliations:** 1 Department of Inorganic Chemistry, Faculty of Pharmacy, Medical University of Gdansk, 80-416 Gdansk, Poland; 2 Department of Physical Chemistry, Faculty of Pharmacy, Medical University of Gdansk, 80-416 Gdansk, Poland; greber@gumed.edu.pl

**Keywords:** antimicrobial peptides, lipopeptides, ESKAPE pathogens, biofilm, microbial resistance, synergism, erythromycin, tetracycline

## Abstract

**Background:** ESKAPE constitutes a group of six nosocomial bacteria that can evade available antimicrobials due to their great potential to develop multi-drug resistance and biofilm-forming abilities. These pathogens often cause hospital-acquired infections and pose a serious threat to public health. The search for efficient innovative therapeutic strategies to fight ESKAPE bacteria have been intensively investigated topics. One promising approach to fight resistant pathogens and their biofilms is combination therapy, which allows the effectiveness against microorganisms to be increased while reducing the applied concentrations and risks of potential unwanted side effects. **Objectives:** The object of the study was to determine if there is an interaction of short lipopeptides ((C_10_)_2_-KKKK-NH_2_, C_16_-KK-NH_2_) together with erythromycin and tetracycline against pathogens of the ESKAPE group (*Acinetobacter baumannii*, *Enterobacter aerogenes*, *Enterococcus faecium Klebsiella pneumoniae*, *Pseudomonas aeruginosa*, and *Staphylococcus aureus*). **Methods:** The checkerboard assay was used to examine the activity of compounds applied in combinations against ESKAPE strains in planktonic cells and toward biofilms formed by *Staphylococcus aures* and *Pseudomonas aeruginosa*. **Results:** The lipopeptides demonstrated a great potential of their application as additives to conventional antimicrobials against Gram-negative bacteria, including microorganisms within biofilms.

## 1. Introduction

ESKAPE constitutes a group of six nosocomial bacteria that can evade popular antimicrobials due to their great abilities for multi-drug resistance (MDR) development. These pathogens include *Enterococcus faecium* (E), *Staphylococcus aureus* (S), *Klebsiella pneumoniae* (K), *Acinetobacter baumannii* (A), *Pseudomonas aeruginosa* (P), and *Enterobacter* spp. (E). Due to their susceptibility to many genetic mutations and the ability to transfer the genes of resistance between species, ESKAPE pathogens were classified by WHO as the pathogens with the highest risk of developing multi-drug resistance (MDR) [1].

The treatment of infections is difficult not only due to the problem of MDR but also the development of bacterial biofilms, which protect the microbes from unfavorable environments, like the presence of antibiotics. Among ESKAPE pathogens, *S. aureus*, *P. aeruginosa* and *A. baumannii* are well-known biofilm formers in healthcare sectors [2]. Bacterial biofilms can grow on various surfaces, including tissues of the human body or biomaterials that are commonly used in numerous therapies. The studies show decreased efficacy of many antimicrobials against these well-organized 3D structures. The determined active concentrations against biofilms can be up 1000 times higher than the concentrations effective against their planktonic counterparts. Biofilm resistance results from several features like the presence of exopolysaccharide, limited access of drugs to microbial cells, alterations in genes connected with antibiotic resistance, and slow metabolism of cells inside the biofilm [3]. The vast majority of chronic infections accompanying the use of biomaterials, like cardiac implants, catheters, and vascular and orthopedic prostheses, result from the formation of biofilms on their surface [4]. Moreover, the biofilm-associated infections often return after the withdrawal of antibiotics. It was confirmed that the presence of a population of microbial cells with reduced metabolism, or ‘persister cells’, allows repopulation of the biofilm after the end of antibiotic treatment [5]. The fact that ESKAPE pathogens often constitute etiological factors of hospital-acquired infections, together with their high levels of resistance to many available antimicrobial agents, makes it difficult to fight them properly. MDR development along with biofilm-associated infections are major causes of treatment failures, causing additional mortality and increases in healthcare costs. Therefore, there is a great need to work on novel effective methods for their eradication.

Short synthetic lipopeptides were designed in order to imitate antimicrobial properties of antimicrobial peptides (AMPs). AMPs are produced by humans, as well as numerous other living organisms, where they participate in the immune defense system against infections [6,7,8,9]. They demonstrate a broad spectrum of antimicrobial activities which includes Gram-positive and Gram-negative bacteria, fungi, and viruses [10,11,12]. Apart from their direct antimicrobial action, AMPs are also immune response boosters, showing the ability to neutralize the action of lipopolysaccharide. Moreover, some AMPs are participating in wound healing processes [13,14]. There are numerous reports demonstrating promising results for AMPs tested against MDR pathogens and biofilms resistant to antibiotics. Moreover, the compounds show relatively low abilities to induce the bacterial resistance development. Despite all the promising features of AMPs, their application in therapy is endowed with many limitations, like potential toxicity, enzymatic degradation, and high costs of production [15,16,17]. There are various approaches focusing on the optimization of biological properties of peptides, as well as improvement of the efficacy of their purchase. Both above-mentioned goals are meant to be achieved by the design of short cationic lipopeptides, which demonstrate activities against drug-resistant microbes as well as effectiveness against biofilms. They are obtained by acylation of short cationic residue and demonstrate surface-active properties due to the presence of the positive net charge and amphipathicity. The synthesis or lipopeptides is less cost- and time-consuming compared to their natural antimicrobial counterpart. However, due to membrane-related mechanisms of action, the lipopeptides also show toxicity toward eukaryotic cells [18,19].

One of potential strategies to fight resistant pathogens and their biofilms is the use of combination therapy based on the combination of two or more antimicrobials to achieve better microbiological activity, a broader spectrum, and higher efficiency. Moreover, combination therapy also allows the application of compounds at lower concentrations and reduces the risks of potential side effects [20].

The aim of the study was to test the activity of two short synthetic lipopeptides (C_10_)_2_-KKKK-NH_2_ and C_16_-KK-NH_2_ applied in combinations with erythromycin and tetracycline against ESKAPE pathogens. The chosen lipopeptides have shown excellent antimicrobial activities in the previous studies, which were comparable or higher than native antimicrobial peptides. Moreover, their synthesis was less time- and cost-consuming in comparison with AMPs [21,22].

## 2. Results

### 2.1. Activity Against Planktonic Bacteria

**Figure 1:**
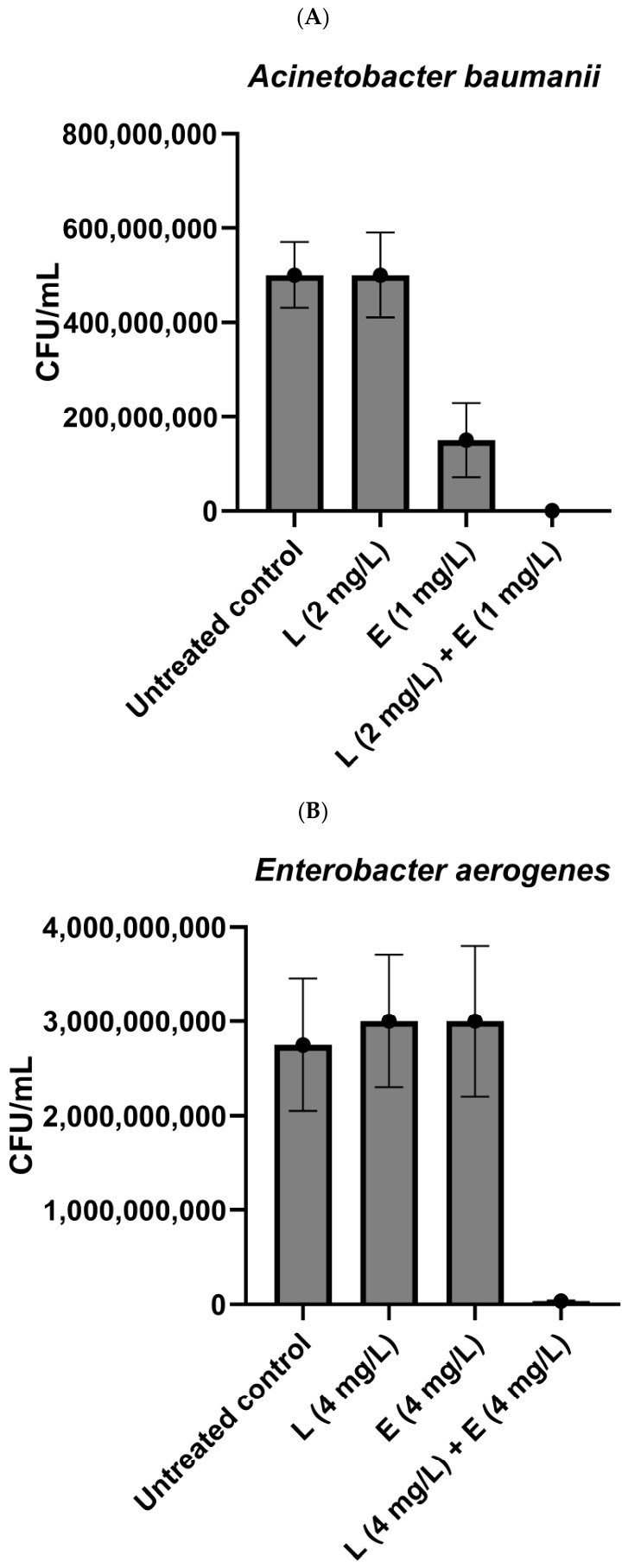

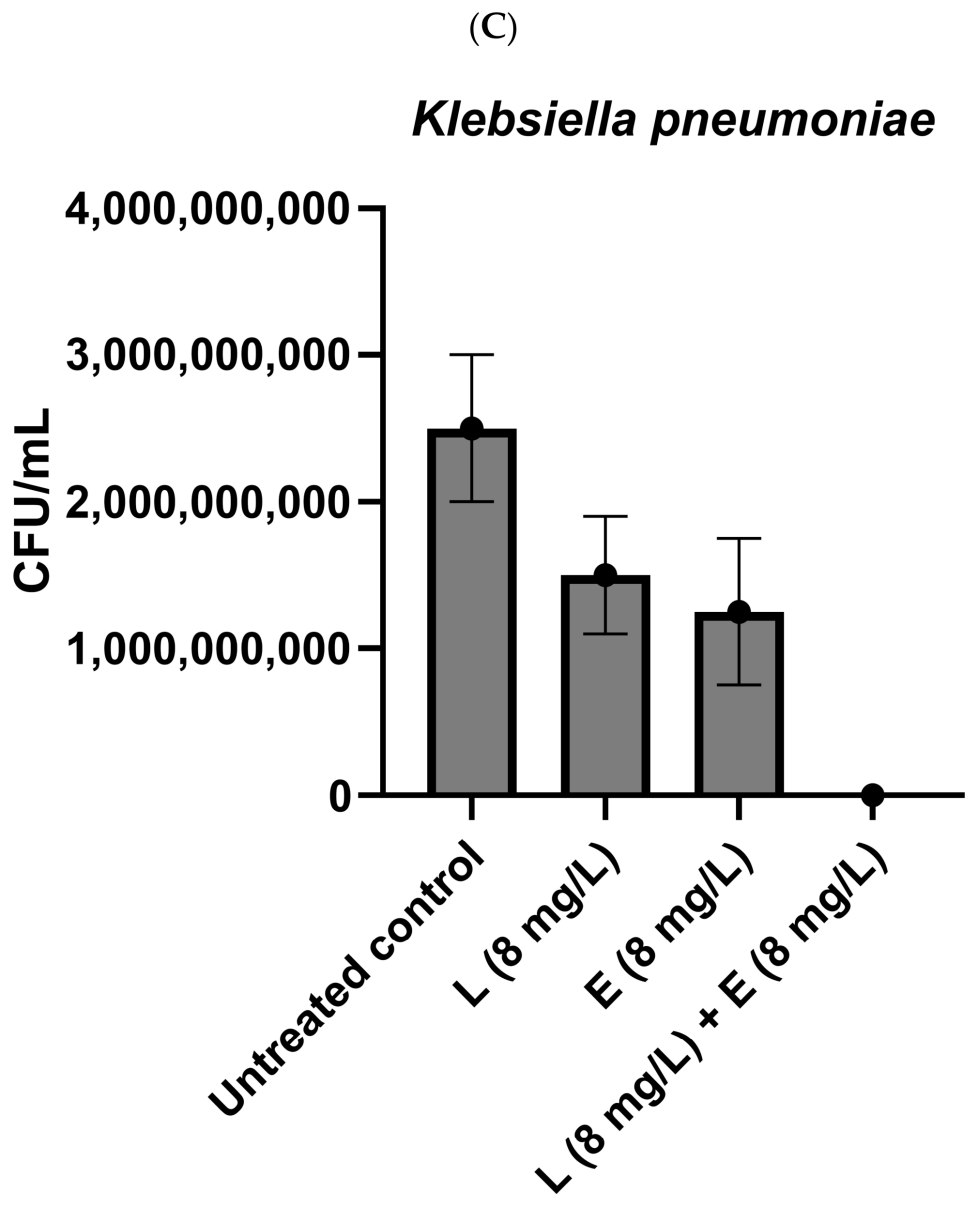
Bacterial growth [CFU/mL] ((**A**)—*Acinetobacter baumanii*; (**B**)—*Enterobacter aerogenes*; (**C**)—*Klebsiella pneumoniae*) after application of lipopeptide (C_10_)_2_-KKKK-NH_2_ (L) and erythromycin (E) applied alone and in combinations at their fractional inhibitory concentrations.

Short lipopeptides applied alone were highly active against *Staphylococcus aureus* (SA) and *Enterococcus faecium* (EF) (8 mg/L, Table 1). Among Gram-negative bacteria, *Pseudomonas aeruginosa* (PA) was the most sensitive to the compounds. The lipopeptides inhibited its growth once applied at a concentration of 16 mg/L. Against the remaining pathogens, the lipopeptides presented moderate or weak activities. Lipopeptide C_16_-KK-NH_2_ inhibited the growth of *Acinetobacter baumanii* (AB), *Enterococcus aerogenes* (EA), and *Klebsiella pneumoniae* (KP) at a concentration of 64 mg/L. (C_10_)_2_-KKKK-NH_2_ demonstrated significantly weaker activity—the compound was active against AB at a concentration of 128 mg/L, while EA and KP were inhibited once the lipopeptide was used at the highest concentration tested (256 mg/L). However, in the case of these two strains, there was a strong synergistic action between the compounds and both antibiotics. The lowest FIC index was calculated in the case of (C_10_)_2_-KKKK-NH_2_ with erythromycin, which also presented weak activity once applied alone (Table 1). Application of this combination resulted in significant reduction in CFU/mL (1.5–3.5 × 10^6^) as compared to the exposure to the compounds alone (1.25–3 × 10^9^) and the untreated control (2.5–2.75 × 10^9^) (Figure 1) Strong synergism between (C_10_)_2_-KKKK-NH_2_ and erythromycin against AB was also observed. This combination turned out to act bactericidal against AB. After 24 h of incubation, the number of bacteria in the samples dropped to 0 (Figure 1). For the combination of (C_10_)_2_-KKKK-NH_2_ with tetracycline against AB and with both antibiotics against PA, a significant decrease in active concentrations was noticed. However, after the calculation of the FIC index, the interaction was classified as additive. Additive action was also demonstrated by C_16_-KK-NH_2_ applied in combination with both antibiotics against AB and with erythromycin against EA and KP, while in the case of tetracycline a synergistic action was observed. In the case of PA, additive action was observed for lipopeptide and tetracycline, while the FIC index obtained for the combination of C_16_-KK-NH_2_ with erythromycin applied against PA classified the interaction as indifferent. No synergistic activities were observed once the combinations were tested against Gram-positive strains. For both strains and the majority of applied combinations, the FIC index indicated additive interactions (Table 1).

### 2.2. Hemolytic Activity

**Figure 2:**
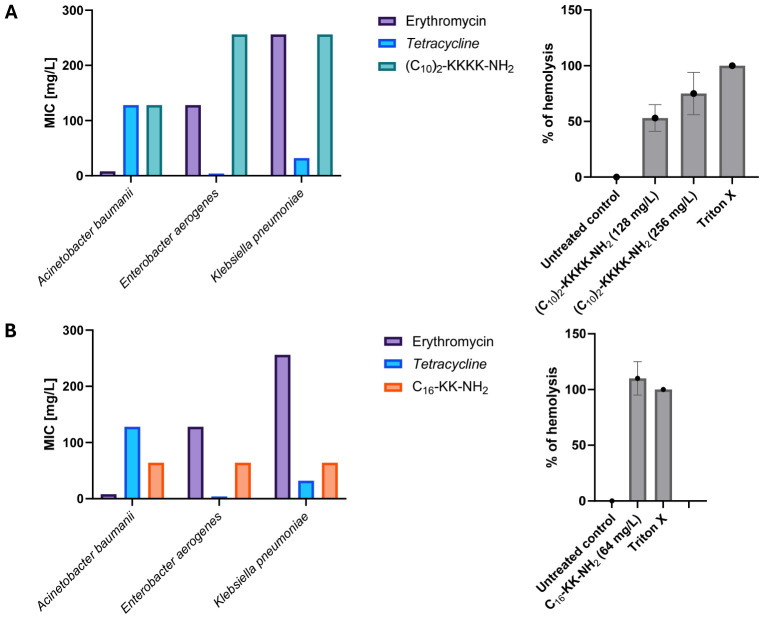

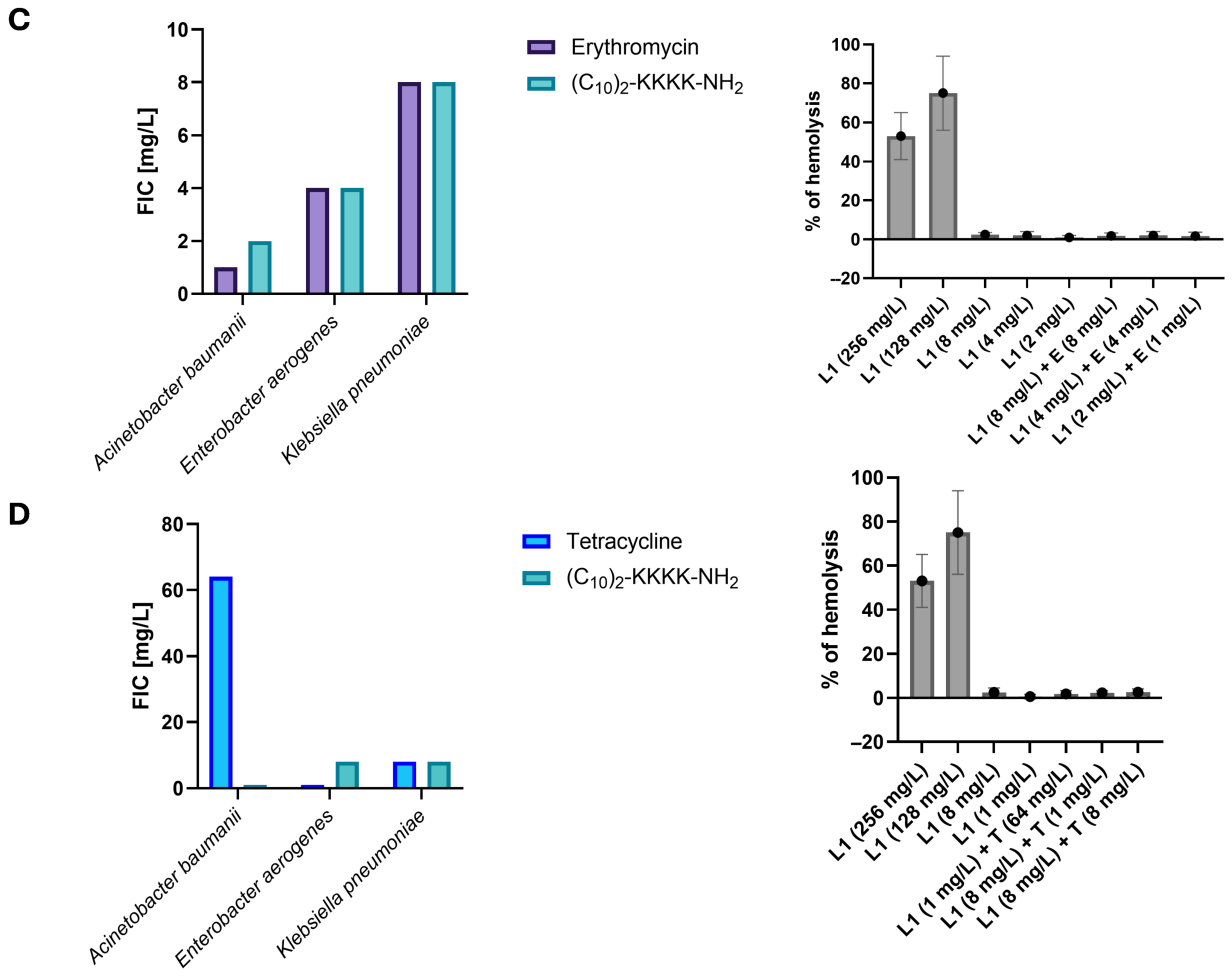

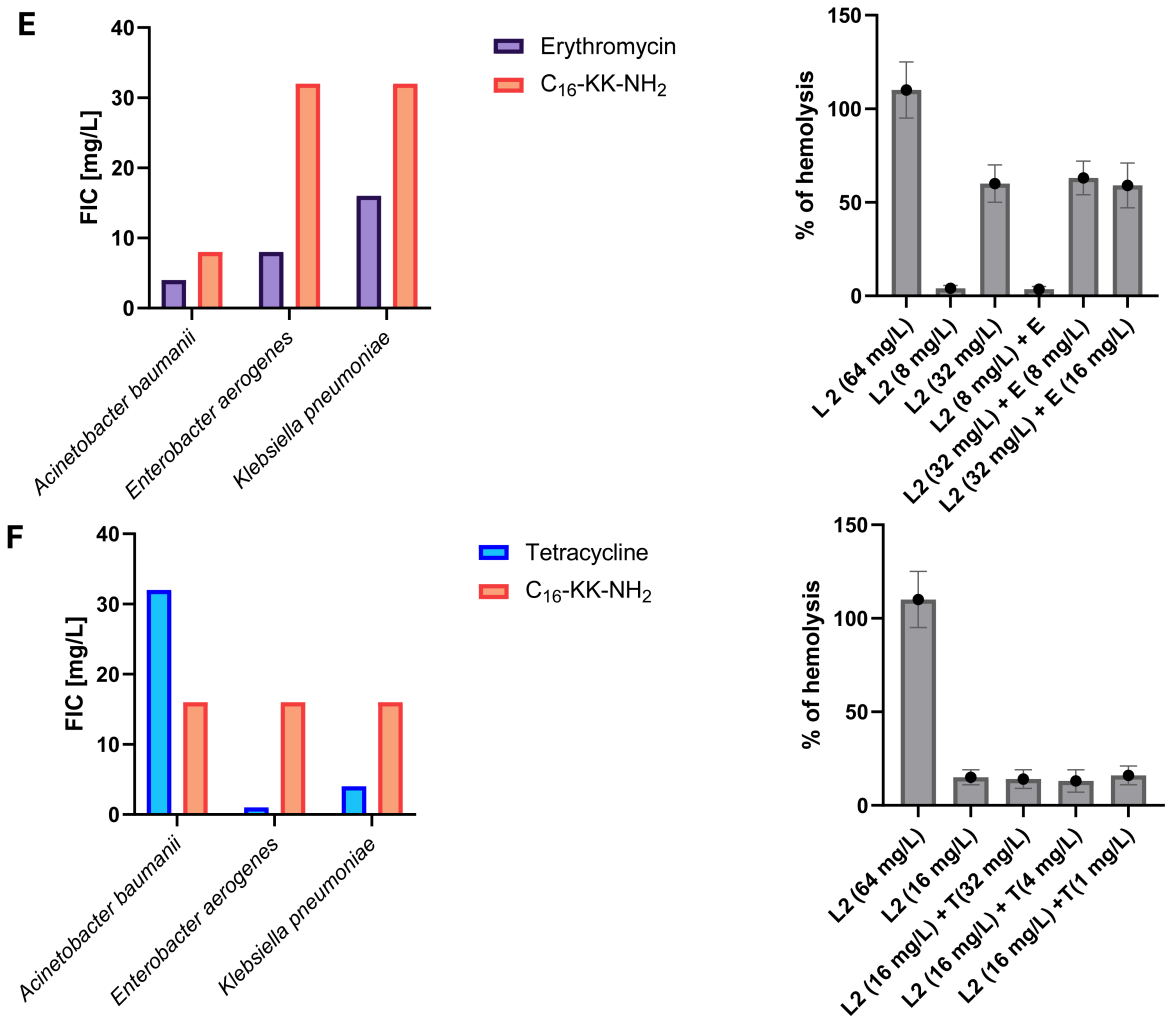
Minimum inhibitory concentrations obtained for lipopeptides L1—(C_10_)_2_-KKKK-NH_2_ (**A**) and L2—C_16_-KK-NH_2_ (**B**) alone; and fractional inhibitory concentrations obtained for combinations of lipopeptides with antibiotics ((**C**)—L1 with erythromycin; (**D**)—L1 with tetracycline; (**E**)—L2 with erythromycin; (**F**)—L2 with tetracycline) and hemolysis caused by the exposition of red blood cells to lipopeptides at determined MICs and FICs.

Considering the fact that cationic lipopeptides show substantial toxicity toward eukaryotic cells [18], the hemolytic activity of synergistic combinations was tested. It was previously reported that the compounds C_16_-KK-NH_2_ and (C_10_)_2_-KKKK-NH_2_ present hemolytic activity toward human red blood cells once applied at a concentration of 16 and 32 mg/L, respectively [21,22]. As expected, the conventional antibiotics did not show potential to disrupt the membranes of sheep erythrocytes, even when applied at the highest concentrations (256 mg/L). Application of lipopeptides in combinations with antibiotics allowed a significant reduction in the active concentrations determined against Gram-negative bacteria. The compounds applied alone were microbiologically active at concentrations found to be strongly hemolytic (Figure 2A,B). The determined FICs were corresponding to the concentrations of lipopeptides which were defined as non-hemolytic for the lipopeptide (C_10_)_2_-KKKK-NH_2_ and less hemolytic in case of lipopeptide C_16_-KK-NH_2_. To exclude the possibility that along with the improvement of antibacterial activity comes the increased toxicity toward eukaryotic cells we have tested the synergistic combinations according to their hemolytic activity. No increase in hemolytic activity was observed for active combinations of (C_10_)_2_-KKKK-NH_2_ and C_16_-KK-NH_2_ with antibiotics. Figure 2C,D presents the fractional inhibitory concentrations determined for lipopeptides on Gram-negative bacteria together with the % of hemolysis after exposure of sheep erythrocytes to the peptides alone and in combinations with antibiotics at their FICs.

### 2.3. Activity Against Biofilms

*Pseudomonas aeruginosa* and *Staphylococcus aureus* were chosen for the biofilm assay as representatives of Gram-negative and Gram-positive strains that are well-known ESKAPE biofilm formers [2]. Short lipopeptides presented weak activity against biofilms formed by PA (Table 2). To eliminate the structures from polystyrene, the compounds were used at a concentration of 128 and 256 mg/L for C_16_-KK-NH_2_ and (C_10_)_2_-KKKK-NH_2_, respectively. Erythromycin was effective against biofilm formed by PA after the application at a concentration of 128 mg/L. Tetracycline eliminated the PA biofilm at the concentration of 64 mg/L. To eliminate the biofilm formed by SA, the antibiotic had to be applied at the highest tested concentration (512 mg/L), while erythromycin turned out to be ineffective across the whole concentration range. The application of combinations of compounds were able to reduce active concentrations in all cases. The lipopeptide (C_10_)_2_-KKKK-NH_2_ demonstrated synergism with both antibiotics once applied against PA. C_16_-KK-NH_2_ exhibited synergistic action with tetracycline, while in the case of erythromycin an additive action was found.

The biofilm formed by SA was eliminated when exposed to lipopeptides at a concentration of 64 mg/L. Conventional antibiotics applied alone turned out to be very weak (tetracycline, MBEC = 512 mg/L) or ineffective (erythromycin) antibiofilm agents. The application of antibiotics in combinations with lipopeptides allowed the significant reduction in the active concentrations of all compounds (Table 2). In the case of erythromycin, the index was not calculated due to the lack of activity of the antibiotic applied alone. However, an undeniable positive influence on the antibiofilm activity was observed. After the supplementation of medium with the lipopeptide (C_10_)_2_-KKKK-NH_2_ at a concentration of 32 and 8 mg/L, the MBECs determined for erythromycin were 1 and 256 mg/L. Application of the lipopeptide C_16_-KK-NH_2_ at a concentration of 8 mg/L caused an antibiofilm effect of erythromycin at 512 mg/L, while the use of lipopeptide at 16 mg/L allowed the concentration of erythromycin to be reduced by half. In the case of tetracycline, the synergistic interaction was demonstrated by both lipopeptides. The combination of antibiotic with (C_10_)_2_-KKKK-NH_2_ allowed the active concentration of lipopeptide and antibiotic to be reduced 4- and 256-times, respectively. In the case of C_16_-KK-NH_2_, the determined MBECs were 2- and 512-times lower in comparison to the MBECs of compounds applied alone. We also observed the antibiofilm activities against SA when tetracycline was used at a concentration of 256 mg/L, with lipopeptides (C_10_)_2_-KKKK-NH_2_ and C_16_-KK-NH_2_ applied at concentrations of 16 and 8 mg/L, respectively. The FBEC index calculated for combinations with tetracycline classified the interaction as additive. However, the results are very promising, as such combinations are able to reduce the concentration of lipopeptides below the values determined as hemolytic.

**Figure 3:**
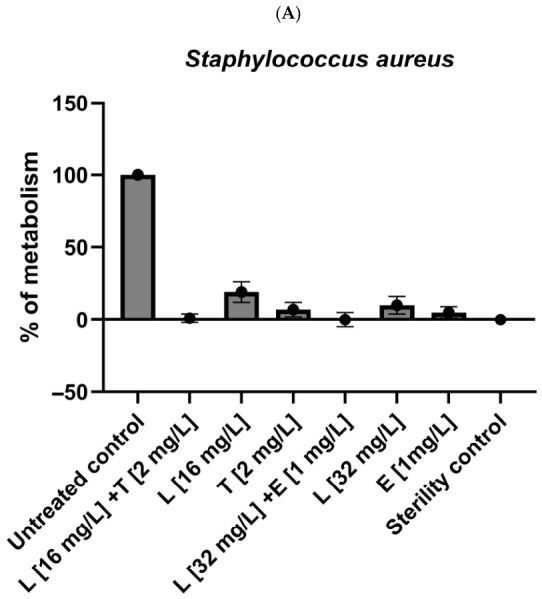

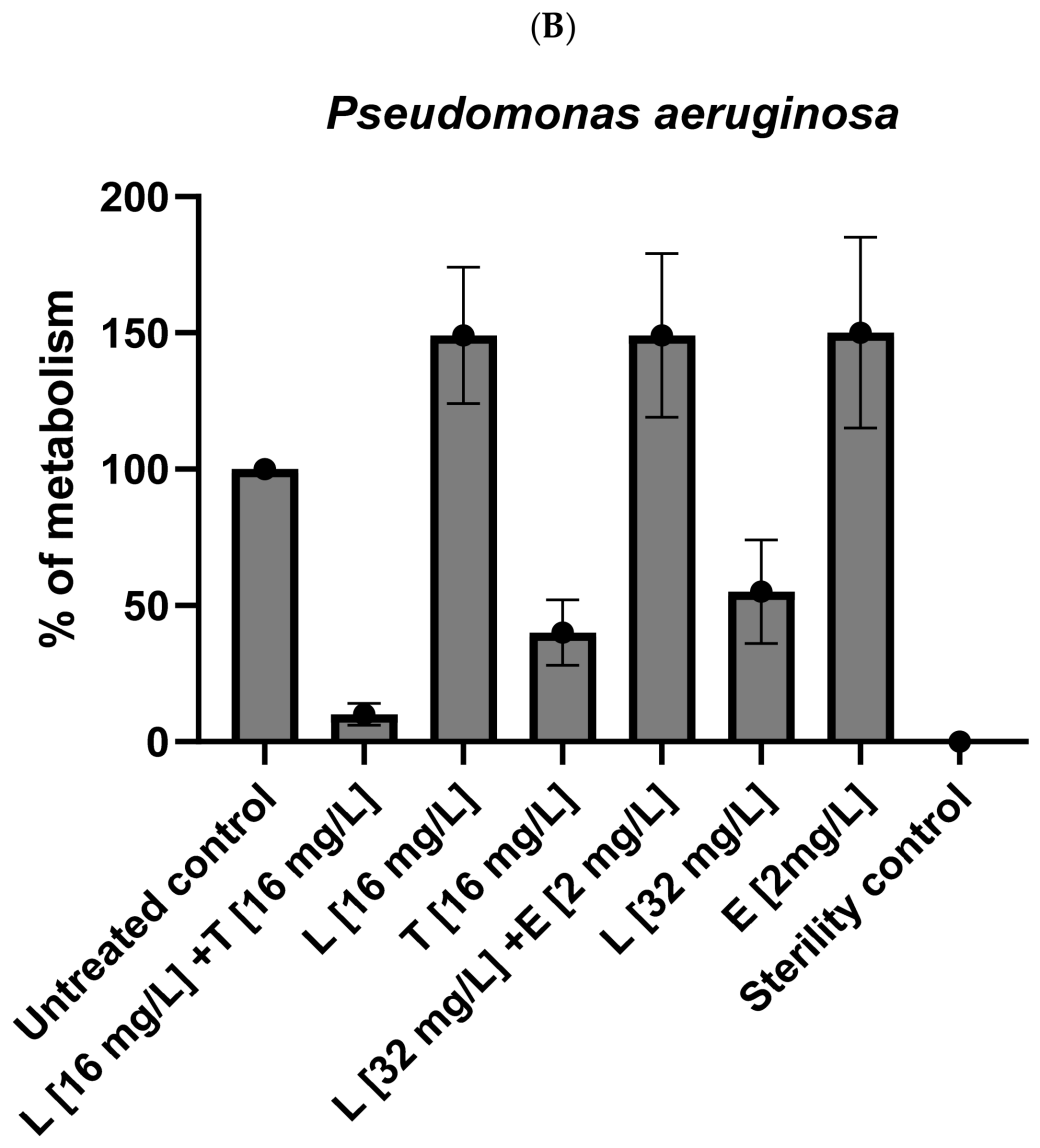
Activity of lipopeptide (C_10_)_2_-KKKK-NH_2_ (L) and its combinations with erythromycin (E) and tetracycline (T) against biofilms formed by (**A**): *Pseudomonas aeruginosa* and (**B**): Staphylococcus *aureus* on PLA materials. The results are presented as the % of bacterial metabolism referred to positive (100%) and negative (0%) controls.

The active combinations of lipopeptide (C_10_)_2_-KKKK-NH_2_ with both antibiotics were further tested against the bacteria cultured on the PLA surface. The results are presented as the % of bacterial metabolism (compared to the untreated control) in Figure 3. The lipopeptide applied with erythromycin not only turned out to be inactive against PA biofilms but also caused a significant promotion of bacterial growth. The metabolism of the treated sample was ca. 1.5 times higher compared to the untreated control. Similar results were obtained when erythromycin and lipopeptide were applied alone at concentrations of 2 and 16 mg/L, respectively. Lipopeptide at a concentration of 32 mg/L and tetracycline at 16 mg/L reduced the metabolism of PA to 40–50%, while the compounds applied together caused bacterial metabolism to drop below 10%. In contrast, the cultures of SA grown on the PLA surface turned out to be more sensitive to the compounds in comparison with the cultures on polystyrene plates. Lipopeptide and both antibiotics caused significant reductions in staphylococcal metabolism once applied alone at the concentrations determined as FBECs on polystyrene plates. However, the application of the compounds in combinations allowed the metabolism to be decreased to the level of the sterility controls.

## 3. Discussion

In the presented study, a synergistic interaction between short synthetic lipopeptides with erythromycin and tetracycline was demonstrated for Gram-negative bacteria, while in the cases of Gram-positive strains, only an additive interaction was observed. However, both lipopeptides applied alone were highly active against SA and EF in their planktonic form. *E. faecium* belongs to physiological flora of the human and animal digestive system, which can cause opportunistic infections. EF demonstrates resistance to many antibiotics, including vancomycin, erythromycin, glycopeptides, tetracycline, aminoglycosides, gentamicin, and streptomycin. It is worth mentioning that the strain used in the study was classified as vancomycin-resistant and demonstrated high levels of resistance to several antibiotics [23,24]. Both lipopeptides were also highly active against staphylococcal biofilms cultured on the polystyrene surface—contrary to erythromycin and tetracycline. The conventional antibiotics inhibited the growth of SA planktonic culture at very low concentrations, but they demonstrated very weak antibiofilm potential. Interestingly, SA cultured on the PLA surface turned out to be much more sensitive to the compounds applied alone. However, the application of combinations allowed for further reductions in bacterial metabolism. *S. aureus* is a Gram-positive cocci present on the skin and nasal mucosa of many humans, but at certain conditions it becomes an etiological factor of a number of severe infections: skin (wounds) infections, pneumonia, osteomyelitis, food poisoning, sepsis, and toxic shock syndrome [25]. The resistance to different penicillin classes has been reported. Moreover, staphylococcal biofilm-related infections associated with commonly used biomaterials cause numerous therapeutic complications [26]. Various combinations of antibiotics with AMPs has shown to be effective against biofilms. The lipopeptide C_16_-KK-NH_2_ showed synergy with vancomycin in the prevention of biofilm-associated infection in a rat model [27]. Application of daptomycin in combination with lipopeptide C_12_-CKK-NH_2_ reduced the development of resistance in *Enterococcus faecalis* [28]. In our previous study, we observed the positive influence of peptides ((C_10_)_2_-KKKK-NH_2_, (C_12_)_2_-KKKK-NH_2_ and temporin A) on the activity of gentamycin against staphylococcal biofilms. It is worth noting that, similarly to the current study, no synergy between compounds in the FIC assay was observed. The increase in antibiofilm activity and the lack of interaction between peptides and gentamycin against planktonic cultures was also noticed in the case of PA [29]. In the current work, PA was the most sensitive Gram-negative strain when lipopeptides were applied alone. PA is Gram-negative and can cause opportunistic infections in immunocompromised patients. Due to the presence of drug efflux pumps, it shows resistance to carbapenems, fluoroquinolones, and aminoglycosides. The results obtained on PA planktonic cells demonstrated synergism for lipopeptide (C_10_)_2_-KKKK-NH_2_ with erythromycin and additive action with tetracycline, while for C_16_-KK-NH_2_, indifference was determined for both antibiotics. For combinations used against PA biofilms on polystyrene, a significant decrease in active concentrations was noticed. This is especially promising due to the numerous therapeutic complications resulting from the development of PA biofilm-associated infections and increased morbidity and mortality rates worldwide. PA constitutes an etiological factor of nosocomial infections with a strong capacity to form biofilms and develop chronic infections, especially in immunocompromised patients [30]. PA biofilms have been reported as difficult to eliminate with many conventional antimicrobials [31]. In our study, all the compounds presented poor antibiofilm potential against PA once they were applied alone. The use of combinations allowed the efficacy against biofilm formed on polystyrene to be significantly improved. However, the results obtained with PLA surface are less promising. Moreover, the active concentrations remained relatively high. Due to the non-specific mechanism of action of AMPs, there is a relatively high risk of toxicity which constitutes the main limitation of the therapeutic application. Many compounds disrupt the membranes of red blood cells when the cells are exposed to the compounds at concentrations close to their active concentrations against planktonic cells [18]. High toxicity in vitro of lipopeptides containing hexadecenoic acid, presenting excellent antibiofilm activities, was previously reported [22,32,33]. The use of (C_10_)_2_-KKKK-NH_2_ with antibiotics allowed the concentrations to be reduced further below those previously determined as toxic toward human cells—keratinocytes and erythrocytes. Moreover, we confirmed that the application of compounds together does not increase the toxicity toward red blood cells. The current study shows the great potential of lipopeptides as promising tools to boost the effectiveness of conventional antibiotics.

Various combinations of antibiotics with AMPs have shown to be effective in previous studies. Considering the large volume of research on AMPs being carried out, there is still not much information regarding their potential in combination therapy with antibiotics. In our previous work, the application of gentamicin with temporin A and the lipopeptides (C_10_)_2_-KKKK-NH_2_ and (C_12_)_2_-KKKK-NH_2_ demonstrated strong synergistic action toward SA biofilms [29]. It is worth noting that there was no synergy between compounds against planktonic cells. Peptide P-113 substituted with non-nature amino acid residues demonstrated promising antimicrobial activity applied with vancomycin against wild-type *E. coli*, as well as vancomycin-resistant *E. faecium* and *S. aureus* [34]. Protamine and 3-methyl-4-isopropylphenol demonstrated synergistic action against *S. mutans* and *S. gordonii* [35]. Ciandrini et al. reported the synergistic activities between AMPs—citropin 1.1, temporin A, cekropin A, and mellitin, as well as a short lipopeptide C_16_-KGK-NH_2_—used in combinations against MRSA biofilms [36]. Co-immobilization of the lipopeptide C_16_-KK-NH_2_ with DNase I on a polydimethylosilaxane surface prevented the formation of biofilms by SA and PA [37]. Biofilm formation inhibition was achieved by the modifications of various surfaces such as titanium, glass, and silicon with various peptides like lactoferricin GZ3.27, GL13K, SESB2V, bacitracin, hLF1-11, chimeric peptides, and Mel-4 [38,39,40,41].

The results of the present study show the great potential of applying lipopeptides as additives to conventional antimicrobials against drug-resistant bacteria, including microorganisms within biofilms. The compounds applied alone show excellent activities against Gram-positive strains, both in planktonic and biofilm forms. Combinations with antibiotics obtained satisfactory efficacy against Gram-negative bacteria. Strong synergism between compounds against Gram-negative strains allowed their spectrum of activity to be broadened to all ESKAPE pathogens. It is worth noting that the enhancement of antibiofilm activities was observed for combinations which did not demonstrate synergy against planktonic cells. Further research should include studies on biofilms formed by remaining ESKAPE strains, as well as investigations of the active combinations according to their potential in the prevention of biofilm formation. Due to substantial differences in activities, biofilms cultured on polystyrene and PLA should crucially include various materials in the future studies. It is well known that the physicochemical properties of a surface determine its susceptibility to microbial colonization [42,43,44,45,46]. This is a very complex issue influenced by many variables, and it is difficult to determine a simple correlation. However, investigation of this topic could provide crucial information for the successful search for novel active agents or combinations to combat biofilm-related infections. 

## 4. Materials and Methods

### 4.1. Bacterial Strains and Culture Conditions

*Acinetobacter baumanii* (BAA-1605), *Enterococcus feacium* (ATCC 700221), *Staphylococcus aureus* (ATCC 25923), *Klebsiella pneumoniae* (ATCC 700603), *Pseudomonas aeruginosa* (ATCC 9029), and *Enterobacter aerogenes* (ATCC 13048) were purchased from the Polish Collection of Microorganisms (Polish Academy of Science, Wroclaw, Poland). Bacteria were cultured in Brain Heart Infusion Broth (BHIB, Biocorp, Warsaw, Poland) under aerobic conditions at 37 °C. After 24 h of incubation, the cultures were centrifuged (10 min, 2500 rpm), washed with phosphate-buffered saline (PBS, AppliChem, Darmstadt, Germany), and suspended in Mueller Hinton Broth II (MHB II, Biocorp, Warsaw, Poland) to the inoculums appropriate for the described assays.

### 4.2. Antimicrobials

Erythromycin and tetracycline were purchased from Sigma-Aldrich. Lipopeptides (C_10_)_2_-KKKK-NH_2_ and C_16_-KK-NH_2_ were synthesized manually according to the previously described protocol [11,12].

### 4.3. Minimum Inhibitory Concentration (MIC) and Fractional Inhibitory Concentration (FIC)

The MICs and FICs of lipopeptides and antibiotics were determined on reference ESKAPE pathogens with the broth dilution method in 96-well spherical bottom polystyrene plates (PS, Kartell, Noviglio, Italy). Lipopeptides dissolved in MHBII were added to the A wells from columns 1 to 10 of the plate (in row 10 the concentration was 2× higher) and serially diluted from top to bottom of the plate from wells A to G. Then, the solutions of antibiotics in MHBII were added to the 10 wells and serially diluted along the plate from wells 10 to 2 (a more precise description of preparation of serial dilutions is presented in Supplementary Files). The wells of columns 11 and 12 were left for positive and negative controls, respectively. Bacteria at inoculums of ca. 5 × 10^5^ CFU/mL were added to the plates to all rows of columns 1–11 and exposed to graded concentrations of tested compounds and their combinations (range 128–1 mg/L for Gram-negative strains and 32–0.125 for SA and EF). The samples were incubated overnight at 37 °C and the results were read visually. MIC/FIC (mg/L) was taken as the lowest concentration of the antimicrobial or their combination which inhibited the visible growth of microorganisms. All experiments were performed in triplicate.

The fractional inhibitory concentration (ΣFIC) index was calculated according to the formula below:

ΣFIC = FIC A + FIC B

where

FIC A = MIC of antimicrobial A in the combination/MIC of antimicrobial A alone;

FIC B = MIC of antimicrobial B in the combination/MIC of antimicrobial B alone.

The combination was considered synergistic when ΣFIC was ≤0.5 and additive when ΣFIC was >0.5 and ≤1 [20].

The combinations with the lowest (ΣFIC) index were further tested in order to determine the number of bacteria in the samples after treatment with lipopeptide (C_10_)_2_-KKKK-NH_2_ and erythromycin applied alone and in combinations, at concentrations determined as the fractional inhibitory concentration on polystyrene plates. The suspensions of AB, EA, and KP at initial inocula of ca. 5 × 10^5^ CFU/mL were exposed to the lipopeptide and erythromycin at concentrations determined as the FIC (alone and in combinations) for 24 h at 37 °C under aerobic conditions. After incubation, the suspensions were diluted and seeded on Mueller Hinton II Agar (MHA II). After 24 h of incubation, the bacterial colonies were counted. The results are the means of three calculations performed on three different days. The results are presented as the number of colony forming units (CFUs) per 1 mL.

### 4.4. Determination of Hemolytic Activity of Combinations of Lipopeptides with Antibiotics

Hemolytic activity was determined on erythrocytes from defibrinated sheep blood (Graso, Starogard Gdanski, Poland). The antibiotics and lipopeptides were dissolved in phosphate-buffered saline (PBS; AppliChem, Darmstadt, Germany) and distributed on the 96-well V-bottom plate analogically as the FIC assay. Red blood cells were suspended in PBS and added to the compound solutions. The final concentration of red blood cells in the sample was 4%. The samples were incubated for 1 h at 37 °C, then centrifuged (800× *g*, 10 min, 4 °C), and the absorbance of supernatant was measured using spectrophotometer (wavelength of 540 nm, Thermo Scientific Multiskan GO, Thermofisher, Waltham, MA, USA). The results were presented as % of caused hemolysis as compared to positive (100% hemolysis—erythrocytes exposed to 1% Triton X-100) and negative controls (0% hemolysis—the suspensions in PBS). All experiments were conducted in triplicate.

### 4.5. Minimum Biofilm Eradication Concentration (MBEC) and Fractional Biofilm Eradication Concentration (FBEC)

Suspensions of SA and PA at initial inocula of ca. 5 × 10^8^ CFU/mL in BHIB were added to the 96-well flat-bottom plates and incubated at 37 °C for 48 h. Biofilms were exposed to combinations of lipopeptides with antibiotics. The dilutions of compounds were prepared analogically for the FIC assay (Supplementary Files). The wells were washed two times with phosphate-buffered saline (PBS) and MHB II was added. Antibiotics were added to the A wells from columns 1 to 10 columns of the plate (in row 10 the concentration was 2× higher) and serially diluted from top to bottom of the plate from wells A to G. Then, the lipopeptides were added to well 10 and serially diluted along the plate from wells 10 to 2. Wells of columns 11 and 12 were left for positive and negative controls, respectively. Biofilms were exposed to graded concentrations of tested compounds and their combinations (range 512–4 mg/L) for 24 h at 37 °C. After incubation, the medium was aspirated and a solution of 0.01% resazurin (Sigma-Aldrich, St. Louis, MO, USA) in MHB II was added as a cell-viability reagent. Upon contact with living cells, blue resazurin is reduced by dehydrogenases to a pink resorufin. After 1 h of incubation, the results were read visually. The results are presented as the lowest concentrations of compounds, where the color of dye corresponded with the negative control (MBEC) or concentrations of compounds at combinations which obtained the same result. MBECs were determined in triplicate on three different days.

The fractional biofilm eradication concentration (ΣFBEC) index was calculated according to the formula below:

ΣFBEC = FBEC A + FBEC B

where

FBEC A = MBEC of antimicrobial A in the combination/MBEC of antimicrobial A alone;

FBEC B = MBEC of antimicrobial B in the combination/MBEC of antimicrobial B alone.

The combinations were classified as synergistic when ΣFBEC was ≤0.5.

### 4.6. Activity of Lipopeptide (C_10_)_2_-KKKK-NH_2_ and Its Combinations with Erythromycin and Tetracycline Against Biofilms Cultured on PLA

Biofilms were grown on polylactic acid cubes placed in 24-well polystyrene plates. The cubes were printed with the 3D printer Artillery x3 Plus (Artillery Technology Co., Ltd., Shenzhen, China) at 220 °C (plate 70 °C). The shapes were printed at a speed of 120 mm/s using Felix Natural PLS Ingeo 1.75 mm (FELIXprinters, IJsselstein, The Netherlands) filament. The cubes were sterilized with an autoclave (121 °C, 1 atm, 20 min) and placed onto 24-well plates with the suspensions of *Pseudomonas aeruginosa* and *Staphylococcus aureus* (initial inoculums of ca. 5 × 10^8^ CFU/mL) in BHIB. The samples were incubated for 48 h under aerobic conditions at 37 °C. The cubes in pure BHIB served as sterility controls. After incubation, the cubes were washed carefully with PBS and placed into wells with solutions (in MHB II) of lipopeptide and antibiotics applied alone and in combinations, at concentrations determined as the fractional biofilm eradication concentration on polystyrene plates. One sample (as well as the sterility control) was washed with PBS and placed in pure MHB II in order to provide a positive control. The cubes were incubated at 37 °C for 24 h and after that were placed into the wells with the solution of resazurin (0.01%) in MHB II. After 1 h, the results were read using spectrophotometer (wavelength of 570 nm and 600 nm, Thermo Scientific Multiskan GO, Thermofisher). The results are presented as the % of bacterial metabolism referred to positive (100%) and negative (0%) controls. The obtained results are means of three readings. The % of bacterial metabolism was calculated according to the formula below:

MA (%) = (ΔAbs of sample–ΔAbs of negative control)/(ΔAbs of positive control–ΔAbs of negative control);

ΔAbs = absorbance at 570 nm–absorbance at 600 nm

## Figures and Tables

**Table 1:** Minimum inhibitory concentrations (MICs) and fractional inhibitory concentrations (FICs) [mg/L] obtained for tested antibiotics and lipopeptides.

	MIC	FIC Lipopeptide	FIC Antibiotic	FIC Index
*Acinetobacter baumanii* BAA-1605				
Erythromycin	8			
Tetracycline	128			
(C_10_)_2_-KKKK-NH_2_	128			
(C_10_)_2_-KKKK-NH_2_ + Erythromycin		2	1	0.14
(C_10_)_2_-KKKK-NH_2_ + Tetracycline		1	64	0.52
C_16_-KK-NH_2_	64			
C_16_-KK-NH_2_ + Erythromycin		8	4	0.625
C_16_-KK-NH_2_ + Tetracycline		16	32	0.50
*Enterococcus feacium* ATCC 700221				
Erythromycin	>128			
Tetracycline	0.25			
(C_10_)_2_-KKKK-NH_2_	8			
(C_10_)_2_-KKKK-NH_2_ + Erythromycin		8	>128	1.0
(C_10_)_2_-KKKK-NH_2_ + Tetracycline		4	0.125	1.0
C_16_-KK-NH_2_	8			
C_16_-KK-NH_2_ + Erythromycin		8	>128	1.0
C_16_-KK-NH_2_ + Tetracycline		8	0.25	2.0
*Enterobacter aerogenes* ATCC 13048				
Erythromycin	128			
Tetracycline	4			
(C_10_)_2_-KKKK-NH_2_	256			
(C_10_)_2_-KKKK-NH_2_ + Erythromycin		4	4	0.05
(C_10_)_2_-KKKK-NH_2_ + Tetracycline		8	1	0.28
C_16_-KK-NH_2_	64			
C_16_-KK-NH_2_ + Erythromycin		32	8	0.56
C_16_-KK-NH_2_ + Tetracycline		16	1	0.50
*Klebsiella pneumoniae* ATCC 700603				
Erythromycin	256			
Tetracycline	32			
(C_10_)_2_-KKKK-NH_2_	256			
(C_10_)_2_-KKKK-NH_2_ + Erythromycin		8	8	0.06
(C_10_)_2_-KKKK-NH_2_ + Tetracycline		8	8	0.28
C_16_-KK-NH_2_	64			
C_16_-KK-NH_2_ + Erythromycin		32	16	0.56
C_16_-KK-NH_2_ + Tetracycline		16	4	0.38
*Staphylococcus aureus* ATCC 25923				
Erythromycin	0.25			
Tetracycline	0.5			
(C_10_)_2_-KKKK-NH_2_	8			
(C_10_)_2_-KKKK-NH_2_ + Erythromycin		4	0.125	1.00
(C_10_)_2_-KKKK-NH_2_ + Tetracycline		4	0.25	1.00
C_16_-KK-NH_2_	8			
C_16_-KK-NH_2_ + Erythromycin		4	0.125	1.00
C_16_-KK-NH_2_ + Tetracycline		4	0.25	1.00
*Pseudomonas aeruginosa* ATCC 9029				
Erythromycin	256			
Tetracycline	32			
(C_10_)_2_-KKKK-NH_2_	16			
(C_10_)_2_-KKKK-NH_2_ + Erythromycin		4	2	0.26
(C_10_)_2_-KKKK-NH_2_ + Tetracycline		8	1	0.53
C_16_-KK-NH_2_	16			
C_16_-KK-NH_2_ + Erythromycin		16	256	2.00
C_16_-KK-NH_2_ + Tetracycline		16	32	1.13

**Table 2:** Minimum biofilm elimination concentrations [mg/L] obtained for lipopeptides and antibiotics applied alone and in combinations (* >512 * no antibiofilm activity observed at tested concentrations; ND * FBEC index not calculated).

	MBEC	FBEC Lipopeptide	FBEC Antibiotic	FBEC Index
*Staphylococcus aureus* ATCC 25923				
Erythromycin	>512			
Tetracycline	512			
(C_10_)_2_-KKKK-NH_2_	64			
(C_10_)_2_-KKKK-NH_2_ + Erythromycin		32 8	1 256	ND * ND *
(C_10_)_2_-KKKK-NH_2_ + Tetracycline		16 4	2 256	0.25 0.56
C_16_-KK-NH_2_	64			
C_16_-KK-NH_2_ + Erythromycin		16 8	256 512	ND * ND *
C_16_-KK-NH_2_ + Tetracycline		32 8	1 256	0.50 0.63
*Pseudomonas aeruginosa* ATCC 9029				
Erythromycin	128			
Tetracycline	64			
(C_10_)_2_-KKKK-NH_2_	256			
(C_10_)_2_-KKKK-NH_2_ + Erythromycin		32	2	0.14
(C_10_)_2_-KKKK-NH_2_ + Tetracycline		16	16	0.25
C_16_-KK-NH_2_	128			
C_16_-KK-NH_2_ + Erythromycin		64	8	0.625
C_16_-KK-NH_2_ + Tetracycline		32	16	0.5

## Data Availability

The original contributions presented in this study are included in the article/Supplementary Materials. Further inquiries can be directed to the corresponding author.

## Supplementary Materials

The following supporting information can be downloaded at: https://www.mdpi.com/article/10.3390/antibiotics15050439/s1, Figure S1: Proceeding of the serial dilution method in the FIC assay: (A) addition of microbiological medium to all 96-wells; (B) addition of lipopeptides to the wells A (from 1 to 9 at a concentration 4 times higher than the final concentrations in the samples and to the well 10 at a concentration 8 times higher than the final concentrations in the samples) and serial dilutions of the well 1 from A to H and of the wells 2 to 10 from A to G; (C) addition of antibiotics at a concentration 4 times higher than the final concentrations in the samples to the well 10 (from A to H) and serial dilutions of the wells A-G from 10 to 1 and the well H from 10 to 2 (D) addition of bacterial suspensions; Figure S2: Tested concentrations of lipopeptides, antibiotics and their combinations in: (A) the FIC assay on Gram-positive strains; (B) the FIC assay on Gram-negative strains; (C) the FBEC assay.

## Abbreviations

The following abbreviations are used in this manuscript:

AMP	Antimicrobial peptide
AB	*Acinetobacter baumanii*
BHIB	Brain heart infusion broth
CFU	Colony forming unit
EA	*Enterobacter aerogenes*
EF	*Enterococcus faecium*
FBEC	Fractional biofilm elimination concentration
FIC	Fractional inhibitory concentration
KP	*Klebsiella pneumoniae*
MA	Metabolic activity
MBEC	Minimum biofilm elimination concentration
MHBII	Mueller Hinton II broth
MIC	Minimum inhibitory concentration
PBS	Phosphate-buffered saline
PA	*Pseudomonas aeruginosa*
SA	*Staphylococcus aureus*
PLA	Polylactic acid
