# Supplementary material for: Short Synthetic Lipopeptides C16-KK-NH2 and (C10)2-KKKK-NH2 Enhance the Activities of Erythromycin and Tetracycline Against ESKAPE Pathogens
Source: Antibiotics (Basel). 2026 Apr 28;15(5):439. doi: 10.3390/antibiotics15050439 (PMC13203588; doi:10.3390/antibiotics15050439)
Supplement: Supplementary file 1 [file antibiotics-15-00439-s001.zip › antibiotics-4102988-supplementary.pdf]

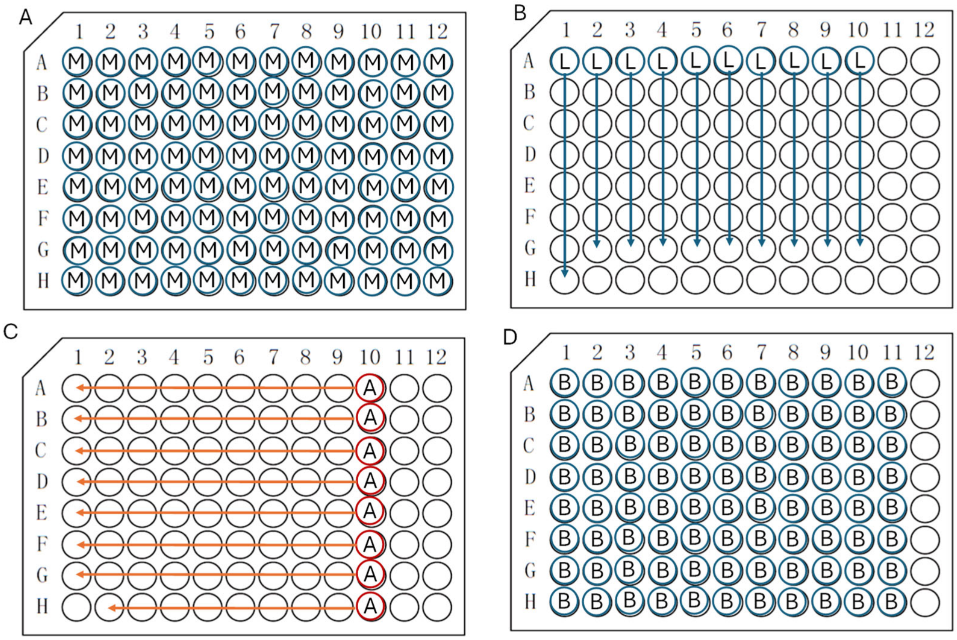

Figure S1. Proceeding of the serial dilution method in the FIC assay: A) addition of microbiological medium to all 96-wells; B) addition of lipopeptides to the wells A (from 1 to 9 at a concentration 4 times higher than the final concentrations in the samples and to the well 10 at a concentration 8 times higher than the final concentrations in the samples) and serial dilutions of the well 1 from A to H and of the wells 2 to 10 from A to G; C) addition of antibiotics at a concentration 4 times higher than the final concentrations in the samples to the well 10 (from A to H) and serial dilutions of the wells A-G from 10 to 1 and the well H from 10 to 2 D) addition of bacterial suspensions

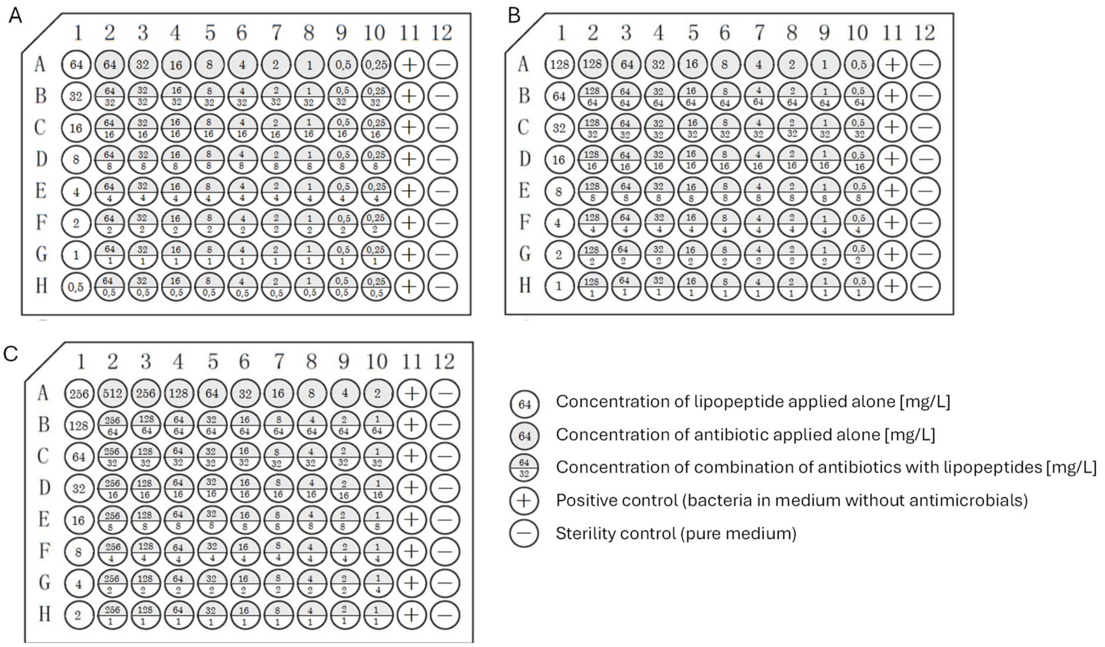

Figure S2. Tested concentrations of lipopeptides, antibiotics and their combinations in: A) the FIC assay on Gram-positive strains; B) the FIC assay on Gram-negative strains; C) the FBEC assay
